# Leveraging Genomic Associations in Precision Digital Care for Weight Loss: Cohort Study

**DOI:** 10.2196/25401

**Published:** 2021-05-19

**Authors:** Ranjan Sinha, Dashyanng Kachru, Roshni Ray Ricchetti, Simitha Singh-Rambiritch, Karthik Marimuthu Muthukumar, Vidhya Singaravel, Carmel Irudayanathan, Chandana Reddy-Sinha, Imran Junaid, Garima Sharma, Patricia Alice Francis-Lyon

**Affiliations:** 1 Digbi Health Los Altos, CA United States; 2 Health Informatics University of San Francisco San Francisco, CA United States

**Keywords:** obesity, digital therapeutics, precision nutrition, nutrigenomics, personalized nutrition, mHealth, mobile apps, gut microbiota, machine learning, health coaching, lifestyle medicine, mobile phone

## Abstract

**Background:**

The COVID-19 pandemic has highlighted the urgency of addressing an epidemic of obesity and associated inflammatory illnesses. Previous studies have demonstrated that interactions between single-nucleotide polymorphisms (SNPs) and lifestyle interventions such as food and exercise may vary metabolic outcomes, contributing to obesity. However, there is a paucity of research relating outcomes from digital therapeutics to the inclusion of genetic data in care interventions.

**Objective:**

This study aims to describe and model the weight loss of participants enrolled in a precision digital weight loss program informed by the machine learning analysis of their data, including genomic data. It was hypothesized that weight loss models would exhibit a better fit when incorporating genomic data versus demographic and engagement variables alone.

**Methods:**

A cohort of 393 participants enrolled in Digbi Health’s personalized digital care program for 120 days was analyzed retrospectively. The care protocol used participant data to inform precision coaching by mobile app and personal coach. Linear regression models were fit of weight loss (pounds lost and percentage lost) as a function of demographic and behavioral engagement variables. Genomic-enhanced models were built by adding 197 SNPs from participant genomic data as predictors and refitted using Lasso regression on SNPs for variable selection. Success or failure logistic regression models were also fit with and without genomic data.

**Results:**

Overall, 72.0% (n=283) of the 393 participants in this cohort lost weight, whereas 17.3% (n=68) maintained stable weight. A total of 142 participants lost 5% bodyweight within 120 days. Models described the impact of demographic and clinical factors, behavioral engagement, and genomic risk on weight loss. Incorporating genomic predictors improved the mean squared error of weight loss models (pounds lost and percent) from 70 to 60 and 16 to 13, respectively. The logistic model improved the pseudo *R*^2^ value from 0.193 to 0.285. Gender, engagement, and specific SNPs were significantly associated with weight loss. SNPs within genes involved in metabolic pathways processing food and regulating fat storage were associated with weight loss in this cohort: rs17300539_G (insulin resistance and monounsaturated fat metabolism), rs2016520_C (BMI, waist circumference, and cholesterol metabolism), and rs4074995_A (calcium-potassium transport and serum calcium levels). The models described greater average weight loss for participants with more risk alleles. Notably, coaching for dietary modification was personalized to these genetic risks.

**Conclusions:**

Including genomic information when modeling outcomes of a digital precision weight loss program greatly enhanced the model accuracy. Interpretable weight loss models indicated the efficacy of coaching informed by participants’ genomic risk, accompanied by active engagement of participants in their own success. Although large-scale validation is needed, our study preliminarily supports precision dietary interventions for weight loss using genetic risk, with digitally delivered recommendations alongside health coaching to improve intervention efficacy.

## Introduction

### Background

The global death toll of COVID-19 has eclipsed 1 million cases [1]. Obesity, following age, has emerged as the most critical risk factor for morbidity, hospitalizations, and complications [2]. The prevalence of obesity in the United States and in other Western countries has increased sharply in the last 2 decades. Since the early 1960s, when more than 10% of Americans were obese, that proportion has grown to 42.4% of adults [3]. Moreover, the prevalence of obesity is higher in minority communities: 49.6% of non-Hispanic Black individuals and 44.8% of Hispanic Americans are obese, compared with 42.2% of non-Hispanic White individuals. These same minority communities are experiencing disproportionate COVID-19–driven mortality, likely linked, at least in part, to the heightened prevalence of obesity [4]. Although the precise cause of obesity is yet to be discovered, several factors have been linked to its development [5]. In particular, biology interacts with behavior and demographics (such as socioeconomic status or ethnic and cultural cuisine) to influence obesity risk [6]. Obesity-associated biological factors include, but are far from limited to, genetics and epigenetics, microbiome composition, age, circadian rhythm disruption, pharmaceutical interactions, and comorbidities and their management [6,7].

The rapid increase in obesity prevalence has coincided with sociological factors, such as generally reduced physical activity alongside a rise in the consumption of highly processed, high-calorie, but nutrient-poor foodstuffs. However, these obesogenic conditions did not affect the population uniformly. Instead, a notable proportion of the population is still able to remain at a healthy weight, indicating that the heterogeneous response to obesogenic conditions may result, in part, from individual innate protection from these conditions, possibly conferred by the genetic makeup [8].

Most current clinical interventions for obesity management focus on lifestyle and dietary adaptation with varying levels of professional guidance and involvement, short- or long-term pharmaceutical therapies, and bariatric surgery [9]. Individual responses to these therapeutic interventions are confoundingly (for clinicians and participants alike) heterogeneous for multifactorial reasons [10], making the need for personalized, precision medicine courses of treatment imperative. Most Americans (63%) have made serious efforts toward weight loss over the course of their lives, and almost one-third are trying to lose weight [11]. In 2014, commercial weight loss services were a US $2.5 billion market consisting primarily of the following market shares—Weight Watchers (45%), NutriSystem (14%), and Jenny Craig (13%)—but the long-term effectiveness of various commercial *calorie restriction*–based weight loss programs is unclear [12-14] (Table 1).

**Table 1:** Commercial weight loss services

Program	Market share (%)	Intervention	Cost per month, US $
Jenny Craig	13	Low-calorie meal replacements with one-on-one counseling	>450
Nutrisystem	14	Low-calorie meal replacements with one-on-one counseling	300-350
Weight Watchers	45	Self-monitoring with web-based coaching and points tracking	43 (plus food)

### Personalizing Weight Loss Interventions

Recent research has elucidated the mechanisms of food-derived biomarkers, allowing for stratification based on a participant’s unique metabolism of given food products. This permits the targeting of personalized nutrition to groups that are better characterized [8,15,16]. For example, given that low-grade inflammation has been implicated in insulin resistance, mediating inflammation via targeted dietary approaches is a precision nutrition intervention [17,18].

Advances have already been made in the early intervention and risk assessment of participants who are obese by designing therapies based on unique genetic predisposition and risk. Environmental interventions such as diet and exercise can trigger epigenetic changes, altering gene expression in metabolic pathways. Recent research indicates that physical activity and high-fat diets may alter DNA methylation patterns in skeletal muscle and adipose tissue [19-21], influencing weight management [8]. Eventually, researchers hope to elucidate the genetic patterns that influence individual obesity and concomitant illness susceptibility, risk of progression, and response to therapy, to provide participants with optimal treatment [22].

### Epigenetics and Their Role in Obesity

Even as science illuminates many genetic risk factors of complex metabolic diseases such as obesity and type 2 diabetes [23-27], these genetic variants account for only a fraction of BMI variation [25]. The *missing* heritability of obesity might be at least partially explained by interactions between genetics and environmental factors [28]. In particular, specific gene variants may influence sensitivity to certain environmental factors so that exposure to these factors in susceptible individuals can contribute to disease. As individuals who are obese are characterized by considerable heterogeneity within the spectrum of clinical obesity, supporting gene-diet interaction and precision nutrition in different subtypes of obesity is imperative [29-33].

Bariatric surgery is a weight loss option for participants with severe and complex obesity, for whom dietary interventions or digital therapeutics have been less than successful [34-36]. Genetics may be a significant predictor of weight loss following Roux-en-Y gastric bypass surgery [37], but few genetic variants have been characterized to date [38,39].

### The Role of Diet in Obesity

Although obesity can, in some cases, be linked to excessive appetite and food consumption, these behaviors may have a genetic component, and even food preferences themselves may have a genetic basis [40,41]. For example, the alpha-ketoglutarate-dependent dioxygenase (FTO) locus rs9939609 has been associated with reduced satiety [42], increased caloric and fat intake [43,44], and a propensity to consume calorie-dense foods [43,45]. The TAS2R38 genotype differentiates potential super-, medium-, and nontasters of bitter-tasting thiourea compounds. These different bitter-tasting profiles appear to be predictive of differential dietary preferences, and in particular, nontasters were observed to have higher BMIs [46]. Considered together and alongside other evidence, this research implies that body weight and BMI may be affected by genetic variations in food preferences, tendencies, and eating behaviors. Elucidating how food intake and body metrics are mediated by genetics is challenging because of the difficulty of reproducing results across varying populations and the complexity of identifying causal interactions [47-49]. Research using randomized controlled trials (RCTs) and large sample–sized biobanks with electronic health records will better characterize how diet and genetics interact to mediate health outcomes [50,51].

### The Role of Physical Activity in Obesity

Exercise that can prevent weight gain and promote weight maintenance has been well established through research [52-55]. Evidence suggests that body weight, waist-to-hip ratio, and BMI are significantly associated with adherence to an aerobic exercise intervention [56]. Interestingly, the propensity for exercise appears to be heritable, at least in part, with studies estimating heritability ranging from 9% to almost 80% [57]. MC4R genes appear to be associated with physical inactivity [58]; however, other genes may be associated with adherence and tolerance to physical activity regimens [56].

### Gut Microbiome and Its Role in Obesity

The human gastrointestinal tract hosts millions of commensal micro-organisms comprising the gut microbiome, which acts as a virtual endocrine organ regulating nutrient production and metabolism, satiety, and even energy homeostasis [8,59]. These microbes are intrinsically linked to host health, as they are implicated in nutrient processing and metabolism, pathogen displacement, vitamin synthesis, and body weight regulation [60]. Researchers and clinicians have been studying alterations of the gut microbiome in individuals, as perturbations in the gut microbiome appear to underlie the pathophysiology of obesity and associated comorbidities, such as type 2 diabetes and metabolic syndrome [61,62]. Microbiome profiling for nutritional intervention is gaining prominence as a key feature of precision nutrition.

Research on the impact of specific dietary factors on microbiome diversity can guide interventions focused on optimizing gut microbial composition [63]. For example, variation in the lactase (LCT) gene region, associated with response to dairy intake, appears to be associated with the abundance of the gut microbiome Bifidobacterium [64]. In particular, variations in LCT were found to be predictive of the obesity-based modulation of dairy lactose and milk intake [65], indicating that shifts in gut microbiota across LCT genotypes could be tied to the caloric extraction of ingested food [65]. Similar to specific genes, specific bacterial species have also been directly implicated in the etiology of obesity. Methanobrevibacter smithii, for example, can itself metabolize dietary substrates or metabolic byproducts of other bacteria, thereby promoting weight gain [66].

Further evidence ties both an individual’s genetics and diet to microbiome composition because lower microbial diversity appears to be associated with excess weight gain [67]. Even in early childhood, disruptions in the gut microbiome can have a long-lasting influence on adult body weight [68]. Moreover, nutritional interventions such as administering prebiotics and probiotics to manipulate gut microbiota that promote or are refractory to weight loss show potential as obesity interventions but require further study [69]. Weight loss, whether mediated by diet or via bariatric surgery, can alter the gut microbiome in ways that affect the efficacy of various weight loss strategies [70,71]. An interesting feature of bariatric surgery is that it appears to induce obesity-associated gut microbiota to shift toward lean microbiome phenotypes [72].

### Behavioral and Digital Interventions in Obesity

As the obesity epidemic continues to proliferate, new digital programs available on websites or as smartphone apps are being leveraged to promote weight loss [73]. Digital programs are agile in that they can easily be modified to reflect the latest research and best practices in a rapidly changing field; they are more cost-effective than traditional in-person programs and are also more easily scalable, increasing their reach [74,75]. Resources can include activity trackers, videos, logs, device-to-device communication, and third-party app compatibility [76]. In addition, research indicates that remotely administered programs can result in significant weight loss [76-79].

Digital programs have the ability to provide personalization to address the plethora of needs presented by participants [76]. Individuals partaking in such programs are still able to leverage interpersonal relationships. Digital health coaching, for example, allows participants to discuss their weight loss journey via any number of communication platforms [75]. According to research, both in-person and telehealth coaching relationships are effective in motivating overweight individuals to work toward weight loss [80]. In a recent study of more than 600 participants in a smartphone-based weight loss program with a coaching component, participants lost, on average, more than 7% of their body weight, successfully passing the 5% weight loss marker that many in-person programs set [75].

The multifactorial nature of obesity is reflected in the myriad heritable, behavioral, and environmental factors that can lead to obesity risk [47]. The most successful interventions are likely to be those that leverage current findings across the full spectrum of obesity-related risk factors: dietary interventions accounting for both genetic markers of food sensitivity, metabolic predispositions, and behavioral risk as well as those geared toward optimizing gut microbial diversity and composition; physical activity measures taken in consideration of genetic risk profiles; and behavioral modifications undertaken via digital care [59]. The precision nutrition therapy offered by Digbi Health aims to account for these various factors in delivering a personalized course of obesity intervention [59]. In this study, we describe the weight loss of a cohort of participants enrolled in the Digbi Health program, modeling and analyzing genomic, lifestyle and engagement factors that were found to be influential in this cohort. It was hypothesized that weight loss models would exhibit a better fit when incorporating genomic data than when using demographic and engagement variables alone.

## Methods

### Recruitment

For this study, we identified all Digbi Health participants who were enrolled between June 2019 and June 2020, had been in the program for at least 120 days, and had been genotyped by Digbi Health. Sample collection kits were shipped to 443 participants, of which 393 mailed back their samples for processing, thereby yielding a cohort size of 393. Among these participants, 315 individuals self-identified as female, 77 individuals self-identified as male, and one individual declined to state. All participants self-enrolled for the Digbi Health therapy via a large California-based insurance payor wellness program. The qualifying criteria to join the program were BMI >25 kg/m^2^ with a comorbidity (eg, prediabetes, diabetes, cardiovascular disease, or hypertension) or BMI >30 kg/m^2^, regardless of comorbidities. Participants were advised to remain under the care and supervision of their existing physicians and were further advised to notify physicians and other health care providers of their participation in the Digbi Health program. The data set included data from each participant’s first 120 days in the program. This Digbi Health anonymized, retrospective research study was exempted from full review by the Ethical and Independent Review Services West Coast Board, Corte Madera, California, reference 20149-01. All participants agreed to the Digbi Health terms and conditions and privacy policy when enrolling in the therapy.

### Intervention

Digbi Health is a next-generation, prescription-grade, digital therapeutic platform that uses artificial intelligence to analyze genetics, gut bacteria, lifestyle habits, and socioeconomic and behavioral risk patterns to create evidence-based personalized nutrition, fitness, sleep, and stress management programs to reduce weight and reverse weight-related inflammatory gut, musculoskeletal, cardiovascular, and insulin-related diseases. Digital precision care interventions are delivered via web-based or mobile apps to expand the accessibility, safety, and effectiveness of health care. Digbi Health’s individualized program is geared primarily toward individuals who are overweight or obese, with or without a comorbidity, and functions as a weight loss management tool. The therapy is currently covered by a large California-based health insurance payor for their qualifying members through its obesity management wellness platform.

On enrolling in the Digbi Health program, participants were provided with web-based log-in credentials and were mailed a Bluetooth-compatible digital weighing scale and saliva and stool biosampling kits. App usage consisted of daily tracking of weight (via the Bluetooth scale), tracking of dietary intake (uploading photographs of all food items consumed), and tracking wellness-associated metrics (sleep quality and quantity, exercise type and duration, stress and meditation, energy levels, cravings, and recommended foods consumed or avoided).

#### Sample Collection

The individual’s DNA was self-collected using a buccal swab (Mawi Technologies iSwab DNA collection kit, model no. ISWAB-DNA-1200). Saliva DNA extraction, purification, and genotyping using Affymetrix Direct to Consumer Array version 2.0 on the Affymetrix GeneTitan was all performed by the AKESOgen laboratory. The results presented in the genetics section of the report were determined by the number of markers and risk genotypes present in the genomic raw data, the Digbi Health reports were loaded into the app, and coaching was individualized based on participants’ genomic risk factors. Individuals’ gut microbiomes were self-collected via a fecal swab (Mawi Technologies iSWAB Microbiome collection kit, model no. ISWAB-MBF-1200). Sample processing and 16SrRNA-targeted next-generation sequencing were performed at the AKESOgen laboratory. Although the app and coaching are personalized based on participants’ microbiome data, these data were not analyzed in this study and are the subject of a forthcoming research article. The personalized Digbi Health plan was systematically reviewed with the participants in individualized sessions with the health coach over a 4-month period at regular, predetermined, weekly, and biweekly intervals.

#### Genetic Report

The Digbi Health genetic report consisted of two sections: gene nutrition and gene fitness. The gene nutrition report analyzed participants’ genotypes that have been shown to influence nutritional traits, such as diet and weight management, micronutrient requirements, food intolerance and sensitivity, and several other attributes relevant to nutritional well-being. For each of these traits, participants were assigned a *high-*, *medium-*, or *low*-risk score based on the number of risk alleles detected, and health coaches guided interventions based on these potential risks (eg, suggesting someone with high risk for gluten intolerance eliminates dietary gluten or someone with medium risk reduces consumption). The degree of risk associated with any specific single-nucleotide polymorphism (SNP) was determined by the presence of 0, 1, or 2 risk alleles. Several individual SNPs may have contributed to a single trait or function, and some of these SNPs might have increased the risk for a trait, whereas others may have decreased it. In Digbi Health gene reports, as many SNPs as possible were considered when determining the risk of a particular trait. Although Digbi Health coaching is individualized based on several different traits, a number of notable traits and associated SNPs and how risk factors for these traits inform individualized health coaching have been highlighted in the *Results* and *Discussion* sections of this paper.

The gene fitness report analyzed SNPs studied in conjunction with fitness regimes, exercise motivation, and the ability to develop various types of muscle fibers. This section of the report also analyzed the potential inflammatory response to exercise, including endurance, strength, and flexibility training. As in the gene nutrition section, each trait was assigned a *high-*, *medium-*, or *low*-risk score based on SNP data, and health coaches guided participants through recommendations for healthy exercise.

#### Gut Microbiome Report

In addition to using genetic risk profiles to guide the course of participants’ precision care, the Digbi Health program also analyzed gut microbiome profiles (collected from stool swab sampling) to guide the course of care. However, in this study, we aimed to analyze only the effect of demographics and lifestyle and genomic factors on weight loss; the incorporation of the related microbiome data and how they inform individualized health coaching is the subject of forthcoming analyses from our group, currently in preparation.

#### Lifestyle

The Digbi Health therapy is a 120-day program that uses body metrics, gut microbiome and genetic profiles, and personalized health coaching to manage weight loss. Participants use the Digbi Health app to track 10 key lifestyle and wellness markers (weight, sleep, hunger, cravings, stress, meditation, superfoods, morning energy, foods to avoid, and exercise) on a daily basis and take photos of the food they consume. Each participant is assigned a health coach who works personally with the participant through 12 guided sessions at various intervals to interpret the personalized wellness reports generated from sampling participants’ DNA and gut microbiota. The reports also provide a breakdown of obesity risk based on individuals’ genetic and gut microbiome profiles. The program is geared toward participants losing at least 5% of their baseline body weight by day 90 of the 120-day program. To achieve this goal, the program seeks to nudge participants toward making incremental lifestyle changes focused on reducing sugar consumption, timing meals to optimize insulin sensitivity, reducing systemic inflammation by identifying possible inflammatory and anti-inflammatory nutrients via genetic testing, and establishing a base level of physical activity. The personalized incremental behavioral modifications suggested by the program are designed to reduce inflammation, optimize gut health based on microbiome testing, and most importantly are supported by health coaching and the app to integrate into the participant's lifestyle so as to be sustainable long term. The genetic profile of Digbi Health users identifies several nutrient and food risk factors that have associations with obesity, comorbidity, or inflammatory risk (eg, gluten sensitivity, lactose tolerance, caffeine sensitivity, fatty acid metabolism, blood pressure response to salt or riboflavin intake, or reduced insulin resistance with exercise), and health coaching guidance is tailored specifically to incorporate participants’ risk profiles.

### Statistical Analysis

The data from our cohort of 393 participants over their first 120 days in the Digbi Health personalized digital weight loss program were analyzed retrospectively. Interpretable regression models (linear and logistic) were built, and visualizations generated using R software (R Core Team). Modeling of demographic and behavioral engagement was conducted by fitting 2 linear regression models of weight loss (pounds lost and percentage lost) in this cohort as a function of the variables listed in Table 2.

**Table 2:** Mean demographic and engagement variables overall and by gender.

Variables	Values, mean (SD)	Males, mean (SD)	Female, mean (SD)
Starting BMI	34.77 (6.66)	33.75 (5.36)	35.01 (6.92)
Age (years)	45.06 (12.02)	46.82 (11.58)	44.63 (12.1)
Number of weight entries	146.39 (133.01)	142.62 (110.18)	147.31 (138.16)
Number of food photos posted	115.2 (139.33)	108.64 (115.59)	116.81 (144.65)
Number of coaching sessions completed	4.95 (2.99)	5.17 (2.98)	4.9 (3)

Genomic-enhanced models were built by incorporating 197 SNPs from participant genomic data as predictors, using Lasso regression on SNPs for variable selection, and then fitting a model to the data set after adding the selected SNPs to the previous engagement variables. The 197 genomic variables were from Digbi-curated panels of SNPs associated with obesity, fitness, nutrient metabolism, and inflammatory markers (Table S1 in Multimedia Appendix 1 [81-88]). Each SNP value was encoded for each participant as their number of risk alleles (0, 1, or 2). One participant did not identify gender, therefore was excluded from all models, resulting in 392 observations included in each of the 4 linear regression models.

Success or failure logistic regression models were also fit, with and without genomic data. Genomic variables were similarly selected from the full panel of 197 SNPs using Lasso logistic regression. Success was defined as ≥5% weight loss, failure as weight gain or negligible change of <2 lb (0.9 kg). Removed from this model were observations of participants who were only partially successful, having lost weight but without reaching the milestone of 5% weight loss. This resulted in the inclusion of 251 cohort participants in the logistic models, both genomic-enhanced and demographic and engagement only.

Insignificant variables were removed from each model, resulting in 6 final interpretable models, half containing demographic and behavioral engagement variables only, whereas the remaining 3 were genomic enhanced.

Demographic variables included gender, age, and baseline BMI. Behavioral engagement variables included the number of coaching sessions completed, number of weight entries, and number of food posts (Table 2). As the number of food posts and number of weight entries were highly correlated (Pearson correlation 0.98), each regression model could include one but not both. To incorporate both variables in modeling, the number of food posts was retained as a predictor for the linear models, whereas the number of weight entries was kept as a predictor for the logistic models.

For genomic-enhanced models, SNP variables were imputed to the most frequent value (mode). SNPs with >10% missing information, high (≥0.8) Pearson correlation with another variable, or zero variance were removed, resulting in 124 SNPs remaining for linear and 122 SNPs remaining for logistic model variable selection by Lasso regression. The SNPs with nonzero coefficients after Lasso regularization for that particular outcome variable (pounds lost, percentage weight loss, and successful weight loss) then served as predictors, along with the three demographic variables and two engagement variables (number of coaching sessions completed along with either number of weight entries or number of food posts).

## Results

### Weight Loss

A total of 393 participants were included in this study to describe and model the weight loss of participants enrolled in the Digbi Health program for 120 days. Of these, 80.4% (315/392) were female and 19,6% (77/392) were male, and one participant declined to state. Tables S1 and S2 in Multimedia Appendix 1 provide a full distribution of the baseline variables. A total of 72% (283/393) participants lost weight compared with 10.7% (42/393) who gained weight, whereas for 17.3% (68/393) participants, the weight remained within normal fluctuations (Table S3, Multimedia Appendix 1). A total of 142 participants lost ≥5% of their baseline body weight within the first 120 days. Improvement in both BMI measures and BMI class over 120 days of treatment is evident in Figure S1, with 25.0% of participants having lost enough weight to move to a lower BMI class (Table S4 in Multimedia Appendix 1). BMI class was defined as presented in Table S5 of Multimedia Appendix 1. The distribution of engagement variables, overall and by gender, is presented in Table 2. In our cohort, no significant difference was found in these variables between males and females (Welch two-sided two-sample *t* tests; results not shown). End points by the obesity class are presented in Table S4 of Multimedia Appendix 1.

As hypothesized, the addition of genomic predictors substantially improved the fit of weight loss models. For linear regression weight loss models (pounds lost and percent), the addition of genomic data improved the mean squared error from 70 to 60 and 16 to 13, respectively, whereas the logistic success or fail model improved pseudo *R*^2^ from 0.193 to 0.285.

**Figure 1:**
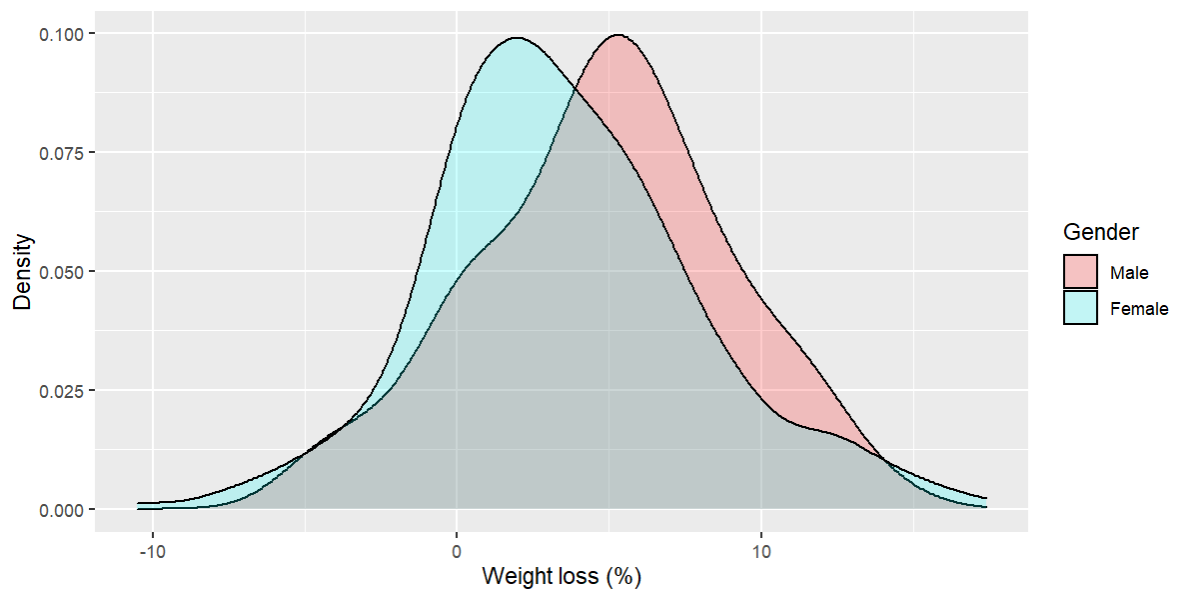
Weight loss (%) distribution by gender.

Figure 1 depicts the distribution by gender of percent weight loss. The difference in percent weight loss for males and females was found to be statistically significant (Welch two-sided two-sample *t* test, *P*=.02). At an average of 4.8 (SD 4.2) percent of body weight lost, the difference in weight loss between males and females was 1.3 (SD 0.5) percent of body weight. Gender was significant to all linear regression models (Tables S6-S9 in Multimedia Appendix 1) but not to the logistic success or fail models (Tables S10 and S11 in Multimedia Appendix 1), as both women and men succeeded in 5% weight loss within 120 days.

### Significant Variables

Unsurprisingly, baseline BMI was significant to both pounds lost linear models (Tables S6 and S7 in Multimedia Appendix 1) but not for any other model. The participant’s age was not significant in any of the models. Increased completion of coaching sessions was significantly associated with increased weight loss in all regression models (Tables S6 to S11 in Multimedia Appendix 1). The two highly correlated engagement variables, number of weight entries and number of food posts, were significant to all models in which they were considered (as described earlier, weight entries were in logistic models, whereas food posts were in linear models; Tables S6 to S11 in Multimedia Appendix 1).

### Significant SNPs

In addition to the demographic and engagement variables described earlier, the genomic-enhanced models identified 10 SNPs that were significant to the linear pounds lost model (Table S7 in Multimedia Appendix 1), 11 SNPs to the linear weight loss percentage model (Table S9 in Multimedia Appendix 1), and 6 SNPs to the logistic model (Table S11 in Multimedia Appendix 1). Of the SNPs found significant to the linear models, 8 SNPs were common in both genomic-enhanced linear models (Tables S7 and S9 in Multimedia Appendix 1). In total, 3 notable SNPs that were found to be strongly associated with changes in body weight for this cohort, rs17300539_G, rs2016520_C, and rs4074995_A, were further explored. The literature suggests explanatory metabolomic factors and findings from recent studies that provide context and explanation for these associations in our descriptive study.

Rs17300539 is located in the promoter region of the ADIPOZ gene, which encodes adiponectin [89]. The high-risk allele has been associated with insulin resistance, whereas the low-risk allele may be associated with protection from weight regain postweight loss intervention [90]. Moreover, the high-risk allele has been associated with higher weight, BMI, and waist and hip circumferences. However, genotype-related differences in BMI became undetectable in the interaction with a diet that is low, below the median (ie, <13% of energy intake) in monounsaturated fats (MUFAs) [91]. This led researchers to propose the possibility of moderating high risk with dietary interventions to reduce MUFAs for those with the risk alleles. Of the 392 participants, 334 were homozygous for the high-risk allele (G), 54 were heterozygous for the risk allele, and 4 were homozygous for the low-risk allele (Table 3).

**Table 3:** Risk allele distribution of highlighted single-nucleotide polymorphisms from weight loss models.^a^

SNP^b^ id	Number of participants, n (%)				
	Risk allele	0 risk allele	1 risk allele	2 risk allele	Missing
rs17300539	G	4 (1.0)	54 (13.8)	334 (85.2)	0 (0.0)
rs2016520	C	244 (62.2)	127 (32.4)	20 (5.1)	1 (0.3)
rs4074995	A	159 (63.3)	67 (26.7)	25 (10.0)	0 (0.0)

^a^Distribution of risk alleles of single-nucleotide polymorphisms from weight loss models that were highlighted in plots and *Discussion* section of the paper.

^b^SNP: single-nucleotide polymorphism.

**Figure 2:**
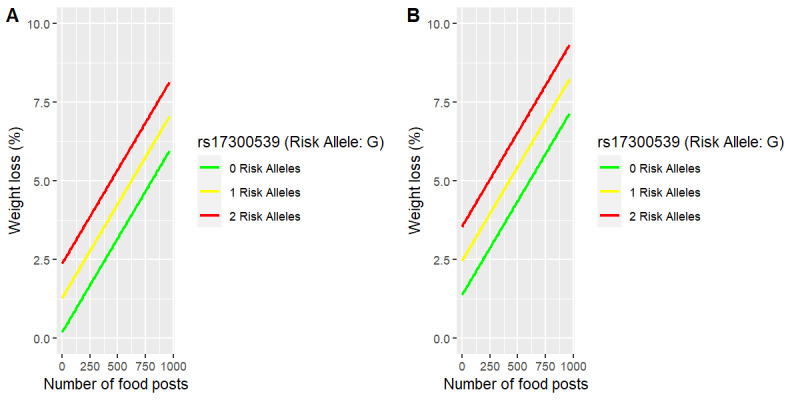
Weight loss (%) versus food posts by rs17300539 (monounsaturated fat intake and weight gain tendency single-nucleotide polymorphism) in females (A) compared with males (B).

The regression models are interpretable models describing weight loss in this cohort and may be visualized to gain insight into variables found to be significant. Figure S3 in Multimedia Appendix 1 and Figure 2 depict the relationships of engagement variables and rs17300539 to weight loss in the genomic-enhanced weight loss percent model. These plots reveal the least squares fit of weight loss percent for females (panel A) and males (panel B), as the two visualized predictors are varied while holding all other model variables constant (SNPs were held constant at their most frequent [mode] values, whereas engagement variables were held constant at their gender-specific means, except for coaching sessions completed, which was fixed at its gender-specific median). The visualizations permit us to see the model relationships of particular predictors, as they impact the outcome variable. For example, the weight loss (%) model fit to this cohort describes the average male having 2 risk alleles who posts no food photos as losing 3.5% of body weight, whereas the average male with the same genomic risk who posts 975 food photos loses 8.75% of body weight (Figure 2). The coefficients of the fitted predictors reveal that in this model, for every 100 additional food posts, participants lose, on average, an additional 0.60% weight while holding all other model predictors constant (Table S9 in Multimedia Appendix 1).

In this model, for each additional risk allele (G) of rs17300539, participants lose, on average, an additional 1.09% weight while holding all other model predictors constant (Table S9 in Multimedia Appendix 1). Similarly, as the number of risk alleles of rs17300539 increases from 0 to 1 to 2, so does percentage weight loss as a function of greater behavioral engagement measured both in the number of completed coaching sessions (Figure S3 in Multimedia Appendix 1) and the number of food photos posted (Figure 2). In essence, participants in this cohort who were at higher risk lost a greater percentage of weight compared with their lower risk counterparts. Moreover, the percentage of weight loss increased in proportion to greater behavioral engagement.

Rs2016520 is a variant of the PPARD gene, which encodes a protein implicated in fat metabolism and baseline cholesterol levels [92]. In women, this SNP has been shown to be associated with muscle development and blood cholesterol reduction after a 12-week exercise regime [93]. High-risk alleles predisposed women to less weight loss on exercise [93]. Of the 392 participants, 20 were homozygous for the high-risk allele (C) of SNP rs2016520, 127 were heterozygous for the risk allele, 244 were homozygous for the low-risk allele, and 1 had no available data (Table 3).

**Figure 3:**
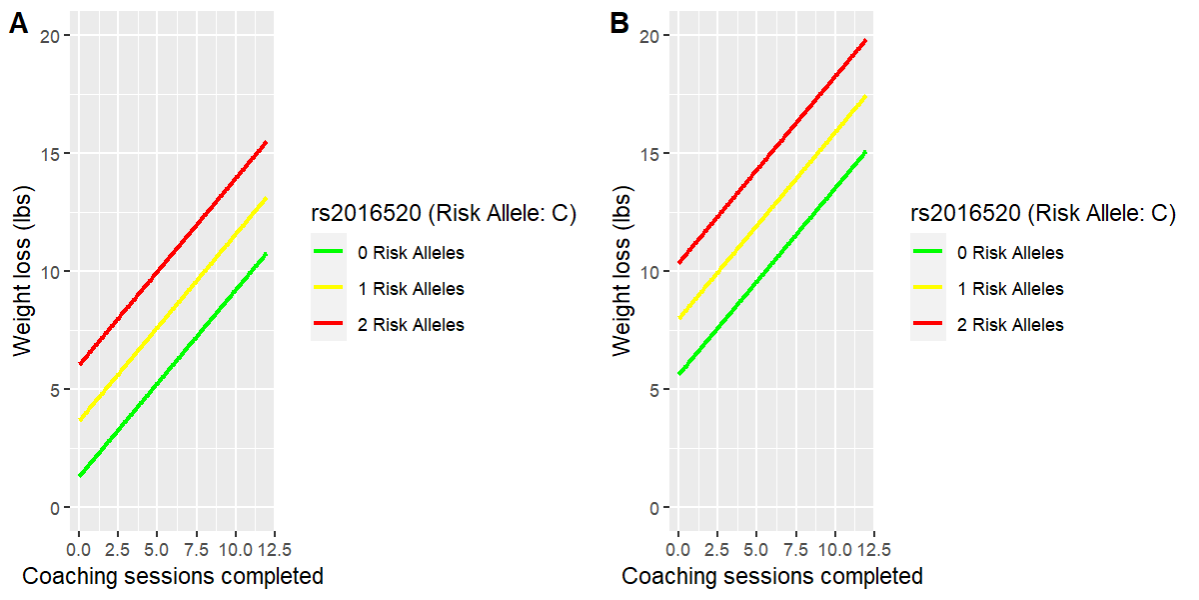
Weight loss (lb) versus completed coaching sessions by rs2016520 (cholesterol single-nucleotide polymorphism) in females (A) compared with males (B).

**Figure 4:**
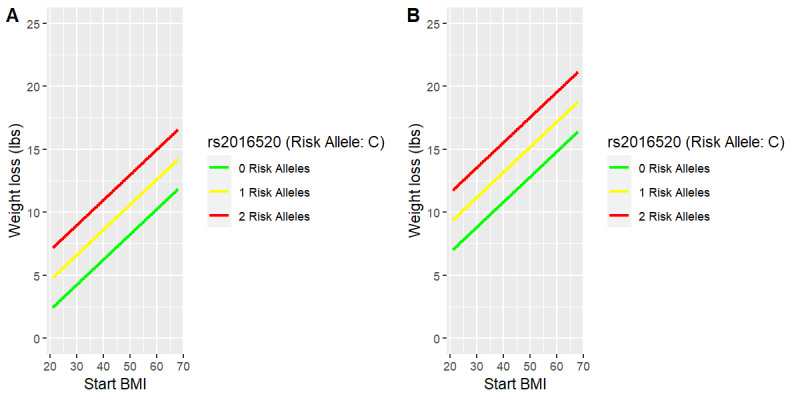
Weight loss (lb) versus baseline BMI by rs2016520 (cholesterol SNP) in females (A) compared with males (B).

Similar to Figure 2 and Figure S3 in Multimedia Appendix 1, Figure 3 and Figure 4 and Figure S4 in Multimedia Appendix 1 reveal the least squares fit of weight loss in pounds for females (panel A) and males (panel B) as the two visualized predictors are varied while holding all other model variables constant. The weight loss pounds model fit to this cohort describes the average female having 2 risk alleles who posts 975 food photos as losing 21 lb (9.5 kg), but only 8 lb (3.6 kg) if no food photos are posted (Figure S4A in Multimedia Appendix 1). Similarly, this model describes the average male having 2 risk alleles as losing 25 lb (11.3 kg) with 975 food posts but only 13 lb (5.9 kg) with no food posts (Figure S4B in Multimedia Appendix 1). As visualized in Figures 3 and 4 and Figure S4 in Multimedia Appendix 1 the effect in this descriptive model of an increase in the number of risk alleles from 0 to 2 is that pounds of weight loss with respect to engagement increases when engagement is measured either as the number of coaching sessions or as the number of food photos posted in the Digbi Health app. Moreover, as seen in Figure 4, a higher baseline BMI was associated with more pounds lost. Males lost more weight than females in each risk group of this SNP.

The rs4074995 SNP has been implicated in calcium-potassium regulation [94]; it is located within the RGS14 gene and is associated with both serum phosphate [95] and serum calcium [96] levels. In particular, each copy of the A allele is correlated with an increase in serum calcium concentration [96]. For the rs4074995_A SNP, of the 251 participants, 25 were homozygous for the high-risk allele (A), 67 were heterozygous for the risk allele, and 159 were homozygous for the low-risk allele (Table 3). The sample size of 251 was smaller than for the abovementioned linear models because rs4074995 was chosen to be highlighted as a predictor of the genomic-enhanced logistic regression (success vs failure) model, which was fit to a subset of the cohort that experienced success, defined as ≥5% weight loss, or failure, defined as weight gain or negligible change of <2 lb (0.9 kg).

**Figure 5:**
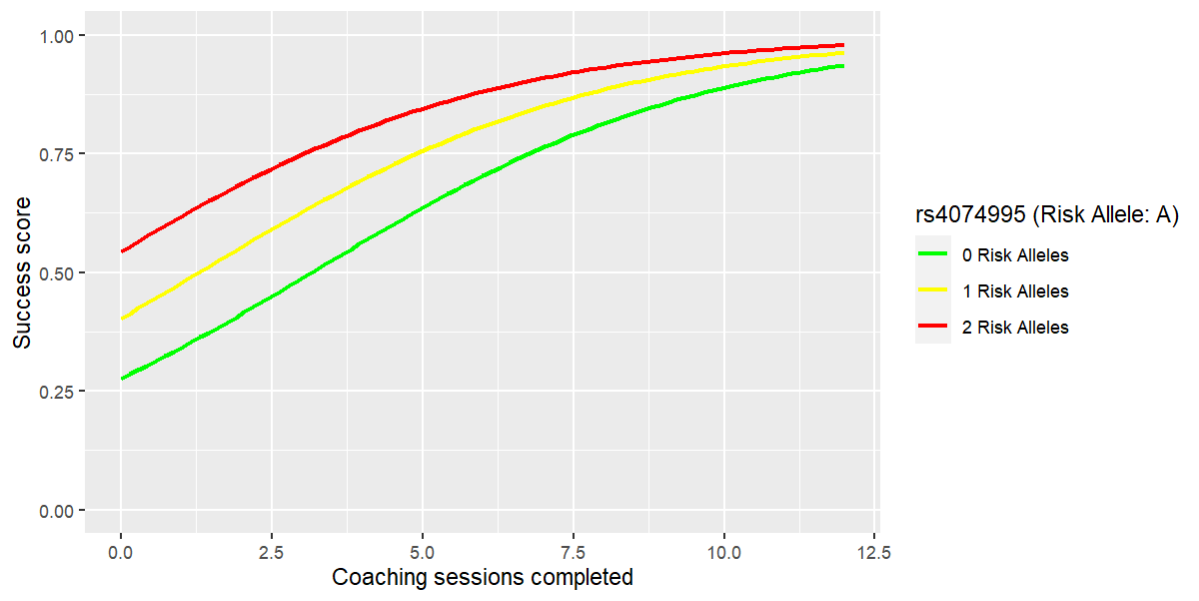
Weight loss success versus completed coaching sessions by rs4074995 (calcium-potassium single-nucleotide polymorphism).

As seen in Figure 5 and Figure S5 in Multimedia Appendix 1, as the risk alleles of SNP rs4074995 increase from 0 to 1 to 2, there is an increase in the success score, which is the likelihood of this model assigning a particular observation to the success class. Similar to the other models, we found that an increased number of coaching sessions completed is associated with a sharp increase in the success score. However, on highest engagement, the effect of risk status diminishes (those with 0, 1, or 2 risk alleles were approximately equally likely to achieve weight loss on highest engagement).

## Discussion

### Principal Findings

A group of 393 participants underwent lifestyle changes over 120 days through the Digbi Health program—a precision digital care program applying machine learning analytics to genetic and microbiome profiles, demographics, and self-reported lifestyle habits—delivering care through the app and weekly health coaching check-ins. Over the duration of the program, patients’ genomic and gut microbiome data pertinent to weight loss (from Digbi Health–curated panels) were provided and translated into lifestyle recommendations and recipes. Of the participants, 72% (283/393) lost weight, whereas 10.7% (42/393) gained ≥2 lb. Of those who lost weight, 50.2% (142/283) were able to lose 5% or more over 120 days.

Interpretable linear regression models of weight loss in this cohort (pounds lost and percentage lost) as a function of demographic and behavioral engagement variables were fit to describe the weight loss of this cohort. Genomic-enhanced models were also built by adding participant genomic data as predictors. Interpretable success or failure logistic regression models were also fit, with and without genomic data. The addition of genomic predictors substantially improved the fit of all models.

The fitted models were examined to gain insights into the weight loss journey of this cohort. Gender, engagement, and specific SNP risk alleles were significantly associated with successful weight loss. The models described greater average weight loss in our cohort for participants having more of certain risk alleles. Here, we consider how successful weight loss may be obtained in the face of greater genetic risk factors. Notably, Digbi Health precision coaching for lifestyle modification is personalized to these genetic risks, and patients reported realizing success that was previously unattainable after being empowered by the knowledge of their genetic and microbiome risk factors, accompanied by advice on lifestyle modifications to address these risks.

We profiled three of these genetic markers (see Results section) to elucidate their relationships with explanatory metabolomic processes and weight gain and loss. Here, we connected these relationships with personalized recommendations delivered by the Digbi Health app and coaching staff. The profiled SNPs were associated with circulating adiponectin and response to dietary MUFA consumption, fat metabolism, and baseline cholesterol levels, and serum calcium levels and calcium-potassium metabolism were strongly associated with weight loss success.

As an example of personalized dietary advice delivered by both the app and coach for program participants who are at genetic risk of weight gain, we considered the advice delivered to participants with a different genetic outlook with regard to rs17300539, a risk allele for weight gain with high MUFA intake. This SNP is depicted in the visualizations of our linear model for weight loss percentage (Figure 2 and Figure S3 in Multimedia Appendix 1). As reported earlier, participants in this cohort who were at higher risk lost a greater percentage of weight compared with their lower risk counterparts, and percentages of weight loss correlated with greater behavioral engagement. This finding can be explained by the fact that those with high risk for this trait were advised by both the app and human coaching to avoid MUFA consumption as much as possible (contrary to the conventional wisdom that these—olive oils, almond oils, etc—are comparatively healthy fats). Instead, they were advised to shift to the consumption of polyunsaturated or saturated fats, depending on their genotypes [97]. Moreover, this SNP is associated with insulin resistance, and parts of the Digbi Health Nutrition Plan (eg, intermittent fasting and reducing processed carbohydrate consumption) would be expected to reduce insulin resistance, addressing the risk associated with this SNP, thereby helping with weight loss [93].

The linear regression pounds lost models found an association between higher baseline BMI and increased weight loss in this cohort (Figure 4). For each one-unit increase in baseline BMI, participants lost an additional 0.2 lb (0.09 kg) on average while holding the other variables in the model constant. This finding could be encouraging to new participants with higher BMI, who may have attempted weight loss with other programs but without much success. Adding genomic information (Figure 3), the model describes the average man of this cohort at the highest baseline BMI, having 2 risk alleles for rs2016520_C and completing five coaching sessions as losing 21 lb (9.5 kg), but women having the same risk outlook and behavioral engagement as losing, on average, 16.5 lb (7.5 kg). When compared with participants of the same gender, baseline BMI, number of coaching sessions, and genomic outlook for all SNPs except rs2016520_C, participants in this data set lost 2.4 lb (1.1 kg), on average, over their treatment for each additional risk allele they had of rs2016520_C (Table S7 in Multimedia Appendix 1).

This SNP is poorly characterized in the general population, but studies associate it with BMI and waist circumference among Han Chinese [98] as well as with cholesterol metabolism [99]. This latter association drives the recommendation by Digbi Health that participants presenting with the high-risk allele limit cholesterol consumption. The association between the number of risk alleles of rs2016520_C and increased weight loss in this data set may indicate the efficacy of data-driven coaching by Digbi Health. A visualization in Figure 3 of this descriptive model depicts a male of average baseline BMI and the most frequent genomic outlook for all except rs2016520_C as losing 20 lb (9.0 kg), on average, if he completed 12 coaching sessions, but only 11 lb (5.0 kg) on average with only one coaching session over the course of treatment.

Calcium is an essential mineral critical for vascular function, muscle function, neurotransmission, cell signaling, and hormone secretion [100]. Serum calcium levels tend not to respond directly to dietary calcium intake, and instead, the body relies on reservoirs in bone tissue to maintain consistent calcium concentrations [100]. Recent research has emerged tying higher serum calcium levels to the development of insulin resistance and cardiovascular hypertension [101]. High serum calcium levels have long been correlated with obesity [102]. Figure 5 depicts the success or fail logistic regression model of the associations between the number of coaching sessions completed and rs4074995_A with successful weight loss while holding all other variables in the model constant at their most frequent number of risk alleles. As with the linear model, in this Digbi Health treatment cohort, increasing total coaching sessions was associated with higher success in losing weight. Those at high risk for excess serum calcium levels were especially encouraged to embrace intermittent fasting and carbohydrate avoidance to combat insulin resistance. This may explain their higher success in achieving ≥5% weight loss (Figure S5 in Multimedia Appendix 1). We found that for participants with more risk alleles of the rs4074995 SNP, success in weight loss increased with more coaching, although it was not as pronounced in those with minimum (0) risk alleles. It may be that success for those with more risk alleles was not as heavily dependent on more coaching sessions, as the app itself conveys pertinent dietary advice.

In addition, our data strongly indicate that behavioral engagement, particularly coaching, contributed to weight loss success. Participants experienced, on average, 0.37% more weight loss with each additional coaching session while holding all other model variables constant (Table S9 in Multimedia Appendix 1). All models found weight loss to be significantly associated with behavioral engagement with the program and app (number of coaching sessions completed, weight entries logged, and food photos logged as predictors). Food photos and weight tracking showed more than 98% correlation with each other and both were significantly associated with weight loss success (Tables S6 to S11 in Multimedia Appendix 1). Prior research has shown that regular engagement with digital weight loss platforms and regular weight tracking is associated with greater weight loss success [103].

We hypothesized that successful weight loss was achieved by adhering to data-driven dietary recommendations that depart from conventional nutritional weight loss advice. Of those 10.7% (42/393) of the participant population who gained weight, there was a notable lack of engagement in the program. Those who gained weight, compared with their counterparts who lost weight, tended to neither engage in coaching nor regularly use the Digbi Health app to log body weight and post food photos of meals. Those who checked in with the coach regularly and logged into the app frequently to post weight and food photos were more likely to lose weight than those who did not.

**Figure 6:**
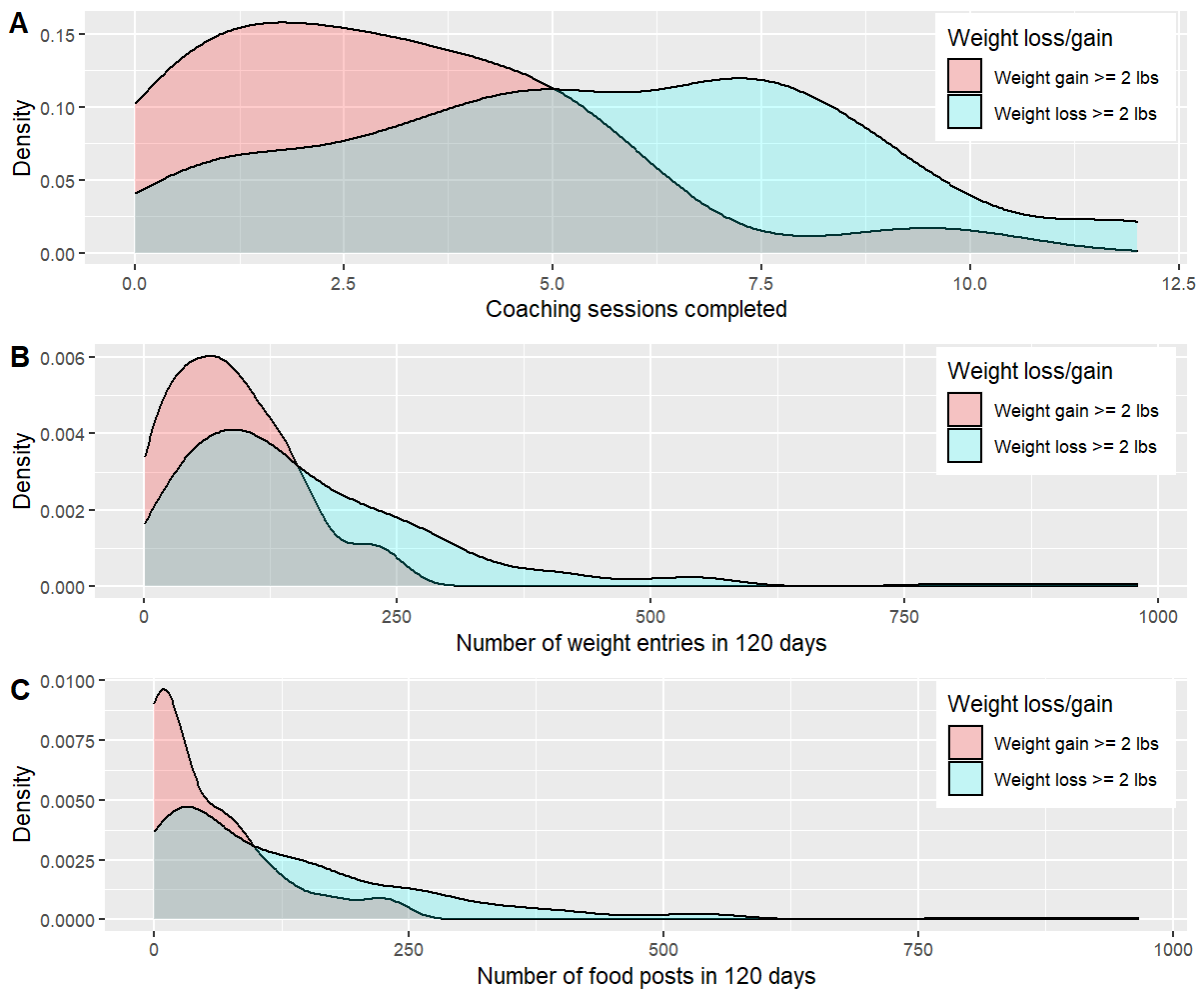
Distributions of engagement variables differ by weight loss group. Measures of engagement were higher in participants who lost weight (blue) versus those who gained weight (red). Statistical difference in means confirmed by the Welch two-sample t test (A) coaching sessions (*P*<.001), (B) number of weight entries (*P*<.001), and (C) number of food posts (*P*<.001). Less than 2-lb gain or loss was considered negligible and excluded from this figure. Engagement variables were summed over the study period of 120 days.

Coaching sessions completed, along with other behavioral engagement variables, differed between participants who lost weight and those who gained weight, whereas baseline weight and BMI did not. The density plots in Figure 6 fairly compare distributions of the two groups: although many more people lost weight than gained, the area under the curve of each group is uniform at 1. Figure 6A illustrates the distributions of completed coaching sessions for those who lost weight (blue) versus those who gained weight (red). The difference is striking: only a fraction of those who failed to lose weight completed at least five (the mean and median) coaching sessions, whereas those who succeeded generally completed five or more. All three measures of engagement were significantly higher in participants who lost weight (blue) versus those who gained weight (red). These distributions are visualized in Figure 6. In contrast, however, Figure S6 in Multimedia Appendix 1 shows no statistical difference in means in (A) baseline weight and (B) baseline BMI, confirmed by the Welch two-sided two-sample *t* test (*P*=.64 and *P*=.42, respectively) between participants who lost (blue) versus gained (red) weight.

Table 2 and Table S1 in Multimedia Appendix 1 show the distributions of variables based on gender. A notable feature of this cohort is that women are grossly overrepresented—a feature that is not specific to the demographics of obesity. Although globally, more women are obese than men, the disparity is driven in large part by demographics, particularly in Africa and the Middle East. In Western countries, men are more likely to be obese [104], which is not reflected in our sample. Instead, our participant demographics may be more reflective of individual self-image. Women appear more likely to perceive themselves as overweight and are more likely to attempt weight loss [105]. Exploration of gender differences in weight loss maintenance reported that men comprised only 27% of participants in behavioral weight loss programs [106,107].

Additionally pertinent to the gender composition of this cohort is that patients self-select and continue with the weight management program of Digbi Health based on the approach they feel works for them. One study indicated that women were more than twice as likely to report having used an organized weight loss program, whereas men were more self-directed in their weight loss [108]. In a single-blinded, randomized clinical trial, the efficacies of three e-coaching approaches were compared: no coaching, nondirected coaching, and directed coaching. Women achieved most success (weight loss, reduction of waist circumference, and improvement of physical activity) in the first 12 weeks with directive e-coaching (similar to Digbi Health), whereas men lost more weight with nondirected e-coaching [80,109]. Men who chose the Digbi Health program succeeded in weight loss. In this cohort, men lost more weight than women over 120 days, consistent with previous findings that men lose weight faster than women [110]. However, the low participation in behavioral weight loss programs, such as that examined here, points to the importance of identifying the underlying factors that impact early engagement and success in weight loss. We are currently undertaking such a study, with the aim of innovating for a level of personalization that will empower and drive success with different subgroups of clients, including men.

Recent research has explored the incorporation of genotypic information into nutritional advice. Researchers have tested the hypothesis that dietary interventions using personalized genotype information have greater efficacy than the same interventions without genomic data, achieving mixed findings [111-115]. However, these studies tested only a few SNPs. Some groups found no benefit from genomic information [22,111], whereas others found specific SNPs (eg, APOE and ACE genes) to be associated with improved dietary changes [21,23]. Some studies observed an increase in positive dietary behaviors on the part of participants who carried a risk allele pertinent to a dietary factor (eg, sodium and fat) with whose recommendations they had not been adhering [21,23]. This aligns with our anecdotal experience of Digbi Health clients, including those in this cohort who had risk alleles for rs17300539, rs2016520, and rs4074995, as described earlier.

Food4Me [111] conducted an RCT to investigate the effectiveness of internet-based personalized nutrition interventions on weight loss and dietary intake with 1269 participants randomized into four groups: one control without personalization and three personalized groups, each with an additional level of personalization: personalized by individual baseline diet, by baseline diet and phenotype (anthropometry and blood markers), and by baseline diet, phenotype, and genotype (5 SNPs). The group comprising participants combined from all three levels of personalized nutrition experienced significantly better improvements in body weight and BMI at month 3 and a more positive behavioral change than the control group. The authors found no evidence that the addition of either phenotype or phenotype plus genotype information to individual baseline diet data enhanced the efficacy of the personalized intervention. However, none of the three personalization interventions, including individual baseline diet, were reportedly tested in isolation for differences in outcomes from the control protocol, and furthermore only five variants were used in genomic personalization.

The five variants used in the Food4Me genomic personalization were from the MTHFR, FTO, TCF7L2, APOEε4, and FADS1 genes [116]. Of these five variants, three were present in the Digbi Health panel of 197 curated SNPs, and of these 3 SNPs, only rs7903146 from the TCF7L2 gene was found to be significant to any of our 3 genomic-enhanced models (linear percent weight loss, linear pounds lost, and logistic success or fail). Rs9939609 (FTO gene) and rs1801133 (MTHFR) were the 2 SNPs that were a part of our gene panel, and hence included as variables, but were found to not significantly contribute to any of the three weight loss models. Rs7903146 was found to be significant to the linear percent weight loss model, and although it was selected as a variable by Lasso for the pounds lost model, it was not statistically significant. Research associates rs7903146 with a higher risk of gestational [117,118] and type 2 [119-121] diabetes as well as reduced insulin levels [119,122]. These medical implications are especially relevant to postmenopausal women [123] as well as those in childbearing years. Unlike the pounds lost model, the percent weight loss model captures weight loss independently of start weight and more aptly models women’s weight loss alongside that of men. Digbi Health has a robust protocol to address diabetes risk and insulin resistance, which differs substantially from the Food4Me personalization based on rs7903146 as reflected in the Food4Me article (including the decision tree for TCF7L2-based information delivered to level 3 *“Diet plus phenotype plus genotype*” in Figure S3 in Multimedia Appendix 1) [111]; thus, we would not expect the outcomes of our personalization regarding this SNP to be the same. We noted that Digbi Health personalization arises from the much broader set of traits in our curated panels and a greater number of SNPs associated with those traits, enabling personalization that is more fine-grained than that informed by a few SNPs. We are not aware of any RCT to test the added value of genomic data that uses the breadth of genotypic markers considered in the Digbi Health program.

### Conclusions

Over the last two decades, the obesity epidemic has coincided with a dramatic change in unhealthy eating habits, a sedentary lifestyle, and physical inactivity. In the United States, more than 40% of the adult population is now overweight or obese. Hereditary predisposition to obesity may have interacted with the obesogenic environment and contributed even further toward the epidemic. The recent accumulation of genomic and lifestyle data has led to the demonstration of possible effects of gene-environmental interactions on obesity [124]. Data from dietary intervention trials indicate that genetic variants, particularly those linked to obesity, metabolism, and nutrient consumption, may significantly alter changes in adiposity and metabolic responses to nutritional interventions and promote effective weight loss [59].

In the foreseeable future, the incorporation of data on genes, eating patterns, metabolites, and gut microbiome into weight loss interventions will be one of the most promising fields of precision care and may allow for the generation of predictable weight loss models based on individual genomic, microbiomic, and metabolomic factors. The goal is precision nutrition, individually tailored to enable effective weight loss and prevent chronic diseases on the basis of genomic history; habitual consumption of food and drink; intake of nutrients (especially those that contribute to disease risks); and metabolomics, microbiome, and other omics profiles of a person [59].

Although using precision medicine to target heterogeneous conditions may seem counter-intuitive, it is the heterogeneous nature of conditions, such as obesity and metabolic illness, that make them such potent targets for intervention, impacting the greatest number of people [8]. Obese subpopulations identified as genetically predisposed to favorably or unfavorably respond to a given weight loss intervention could be targeted accordingly.

To date, few studies have investigated metabolomic functioning, lifestyle and behavioral mechanisms, and gut microbiome, which can affect obesity and health at the interface between genetic variation and the environment. The Digbi Health digital precision weight loss program operates at this interface. This study was limited by its retrospective and descriptive nature. The field of precision nutrition would benefit from additional prospective randomized controlled studies on a larger scale. Although such studies will be needed to validate these findings, the analysis and modeling presented here appear to support dietary precision interventions considering genetic predisposition to disease and genetic variants defining dietary preference and metabolic risk. In addition, our results point to the efficacy of coaching that empowers and actively engages participants in their own success.

Future studies should explore the synergistic effects of genomic variables in interactions with other genome, microbiome, and lifestyle and behavior variables. A follow-up to the work presented here, exploring not only the effect of incorporating genomic data but also including the microbiome data used in Digbi Health precision care, is currently in preparation. Personalized protocols that incorporate data on genes, eating patterns, metabolites, and gut microbiome into weight loss interventions may well be a promising field of precision care, allowing for the generation of predictable weight loss models that account for the synergistic effect of these influential factors.

## Appendix

Multimedia Appendix 1 Distributions of predictors, weight loss outcomes, and results of descriptive models.

## Abbreviations

FTO: alpha-ketoglutarate-dependent dioxygenase
LCT: lactase
MUFA: monounsaturated fat
RCT: randomized controlled trial
SNP: single-nucleotide polymorphism
