# Supplementary material for: Leveraging Genomic Associations in Precision Digital Care for Weight Loss: Cohort Study
Source: J Med Internet Res. 2021 May 19;23(5):e25401. doi: 10.2196/25401 (PMC8173391; doi:10.2196/25401)
Supplement: Multimedia Appendix 1 [file jmir_v23i5e25401_app1.pdf]

Leveraging Genomic Associations in Precision Digital Care for Weight Loss: Cohort Study

Supplementary Material

This is a Supplementary Appendix to a full manuscript published in the J Med Internet Res.
For full copyright and citation information see http://dx.doi.org/10.2196/25401

Table S1: Genomic variables significant to one or more of the 3 models utilizing genomic data

SNP genotype: % participants/males/females with given number of risk alleles												
	Participants: n = 392				Male: n = 77 (19.6%)				Female: n= 315 (80.4%)			
SNP	0 risk %	1 risk %	2 risk %	no value%	0 risk %	1 risk %	2 risk %	no value%	0 risk %	1 risk %	2 risk %	no value%
rs10246939	25.26	47.45	27.30	0.00	22.08	51.95	25.97	0.00	26.03	46.35	27.62	0.00
rs1042713	33.42	50.51	14.80	1.28	37.66	48.05	12.99	1.30	32.38	51.11	15.24	1.27
rs10741657	15.82	41.33	42.86	0.00	14.29	41.56	44.16	0.00	16.19	41.27	42.54	0.00
rs1501299	7.14	45.41	47.45	0.00	5.19	48.05	46.75	0.00	7.62	44.76	47.62	0.00
rs17300539	1.02	13.78	85.20	0.00	0.00	15.58	84.42	0.00	1.27	13.33	85.40	0.00
rs1799931	88.52	11.22	0.26	0.00	93.51	6.49	0.00	0.00	87.30	12.38	0.32	0.00
rs2016520	62.24	32.40	5.10	0.26	57.14	38.96	3.90	0.00	63.49	30.79	5.40	0.32
rs2112347	17.09	43.11	39.54	0.26	23.38	42.86	32.47	1.30	15.56	43.17	41.27	0.00
rs2185570	75.51	22.96	0.51	1.02	75.32	22.08	1.30	1.30	75.56	23.17	0.32	0.95
rs236918	4.34	19.39	76.02	0.26	2.60	18.18	79.22	0.00	4.76	19.68	75.24	0.32
rs4074995	60.71	30.36	8.93	0.00	55.84	31.17	12.99	0.00	61.90	30.16	7.94	0.00
rs7138803	45.92	45.41	8.67	0.00	41.56	54.55	3.90	0.00	46.98	43.17	9.84	0.00
rs7903146	54.08	35.20	10.71	0.00	57.14	37.66	5.19	0.00	53.33	34.60	12.06	0.00
rs9376026	30.36	45.41	24.23	0.00	27.27	50.65	22.08	0.00	31.11	44.13	24.76	0.00

Table S1: In all, 197 genomic variables from Digbi Health's curated panels were examined in this study; those that were significant to one or more models are reported here. These SNPs are not sex-related variants, and no significant difference was detected between males and females (Welch's two-sample t-test with Benjamini-Hochberg correction for multiple hypothesis testing, results not shown).

Table S2 Gender Distribution

Gender	Number of participants	Number of participants (%)
Female	315	80.2
Male	77	19.6
Decline to state	1	0.2

Table S2: Distribution of Digbi Health study participants by gender

Table S3: Weight Change in Study Participants

Weight change status	Number of participants	Number of participants (%)
Gained Weight	42	10.7
Negligible (< 2 lb) change	68	17.3
Lost Weight	283	72.0

Table S3: Distribution of participants by weight change status on Day 120 in Digbi Health program

Table S4 - BMI Shift in Study Participants

Weight change category	Number of participants	Number of participants (%)
Shifted to higher BMI class	7	1.7
No change in BMI class	288	73.3
Shifted to lower BMI class	98	25.0

Table S4: Distribution of participants by shift in BMI class by Day 120 in Digbi Health program

Table S5: Obesity classes of participants, before and after treatment

BMI class	Number of participants in class at program start	Number of participants in class post 120 days
Normal	5	16
Overweight	87	108
Class I	136	133
Class II	91	81
Class III	74	55

Table S5: Obesity classes were categorized into Normal (BMI <25), Overweight (25 <= BMI < 30), Class I (30 <= BMI < 35), Class II (35 <= BMI < 40), and Class III (BMI >= 40).

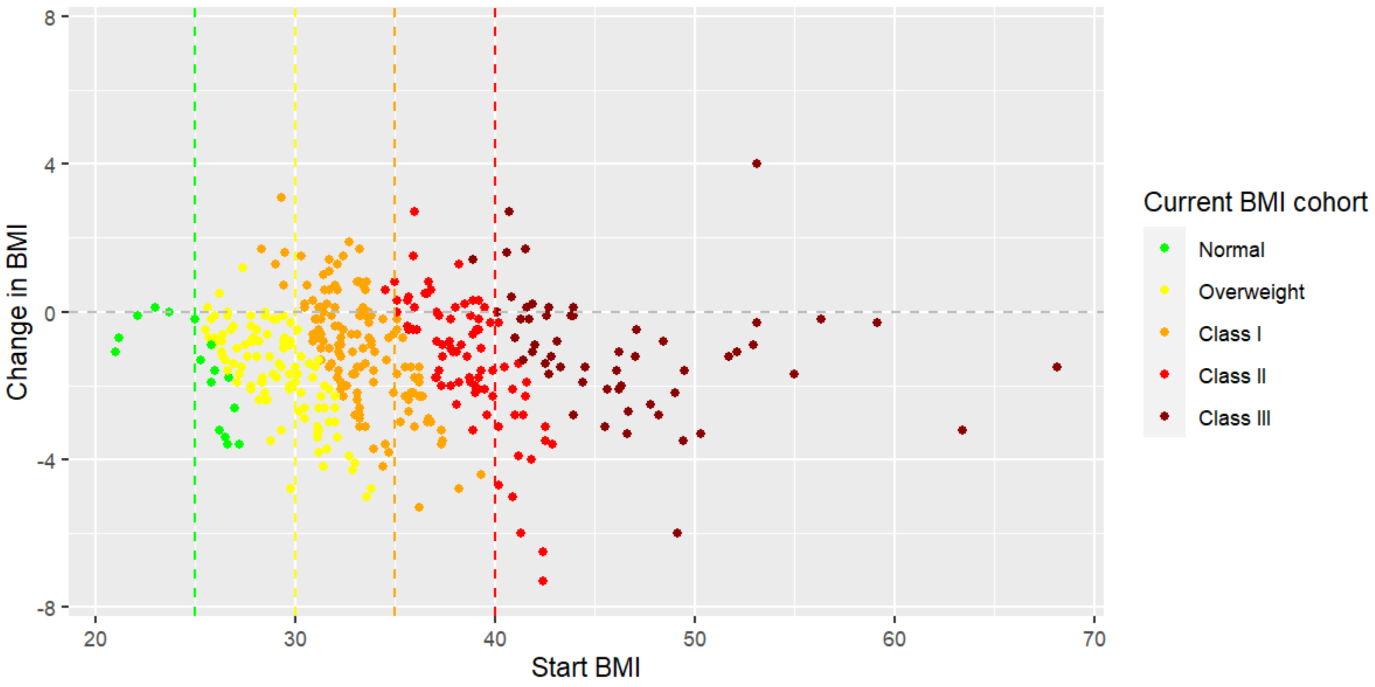

Figure S1: Change in BMI class vs Baseline BMI by Day 120 BMI Class. Post 120 days in the program, a positive shift of number of individuals across BMIs classes was observed. The dispersion of obesity classes based on baseline weight and current weight in the cohort are displayed in Figure S1 and Tables S4 and S5.

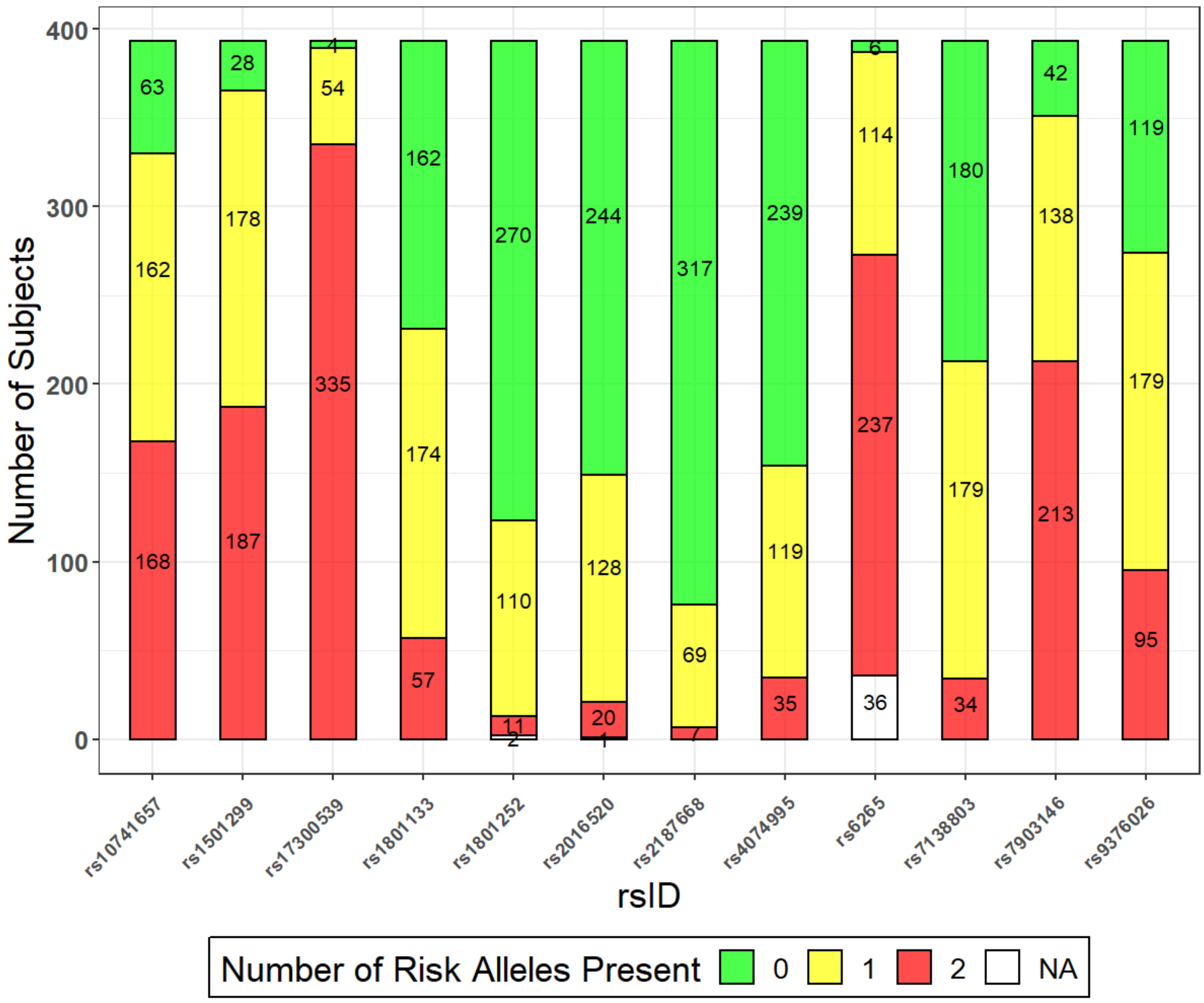

Figure S2: Distribution of Selected SNPs across Digbi Health Subjects

Rs10741657 has been linked to serum concentrations of Vitamin D, with carriers of the G allele having lower levels of Vitamin D [117], rs1501299 is a variant of the ADIPOQ gene encoding adiponectin [118], rs1801133 is in the MTHFR gene which is involved in folate metabolism with A allele implicated in lower enzymatic activity [119], rs1801252 is in the ADRB1 gene whose encoded protein is the target of beta blocker drugs for reducing hypertension [120], rs2016520 is associated with baseline cholesterol levels [84], rs2187668 is related to autoimmune disease and is implicated in both lupus and celiac disease [121, 122], rs4074995 is associated with serum calcium levels [88], rs6265 has been associated with caffeine metabolism profiles [123], and rs7138803 is associated with measures of obesity [124].

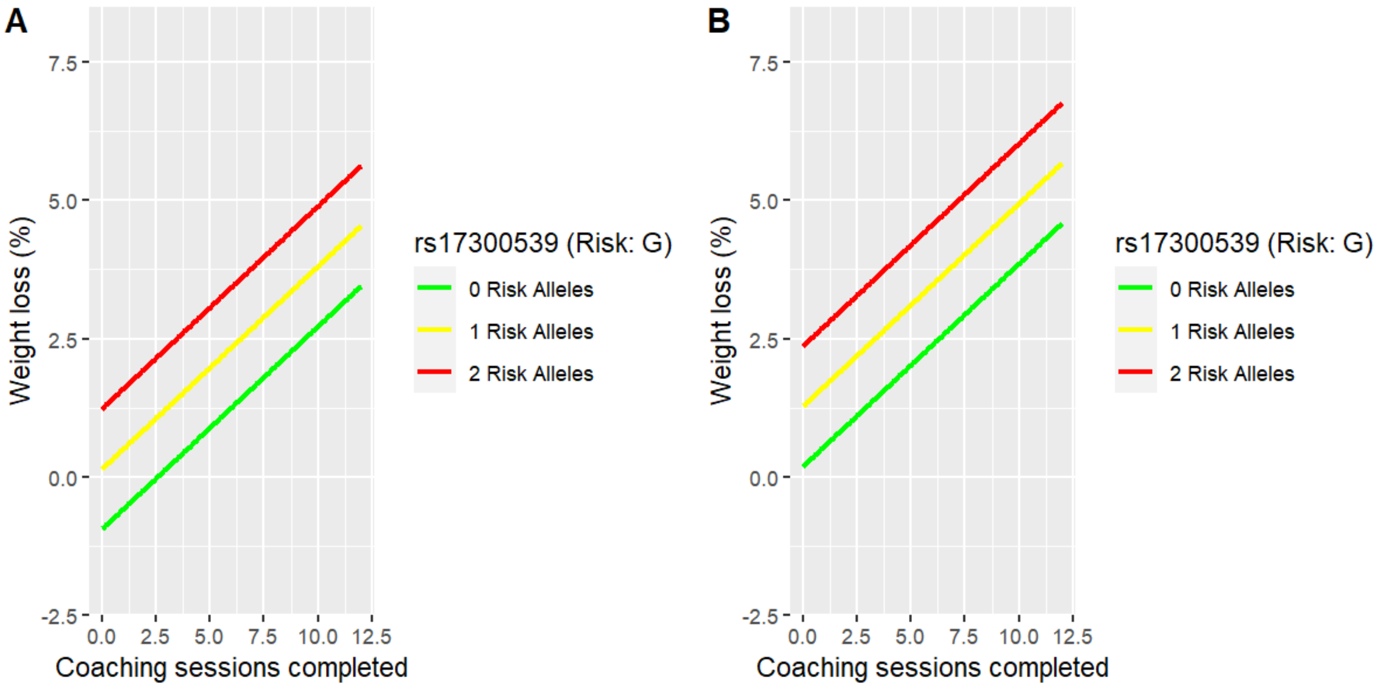

Figure S3: Weight loss (%) vs Coaching by rs17300539 (mono unsaturated fats intake and weight gain tendency SNP) in females (A) compared with males (B).

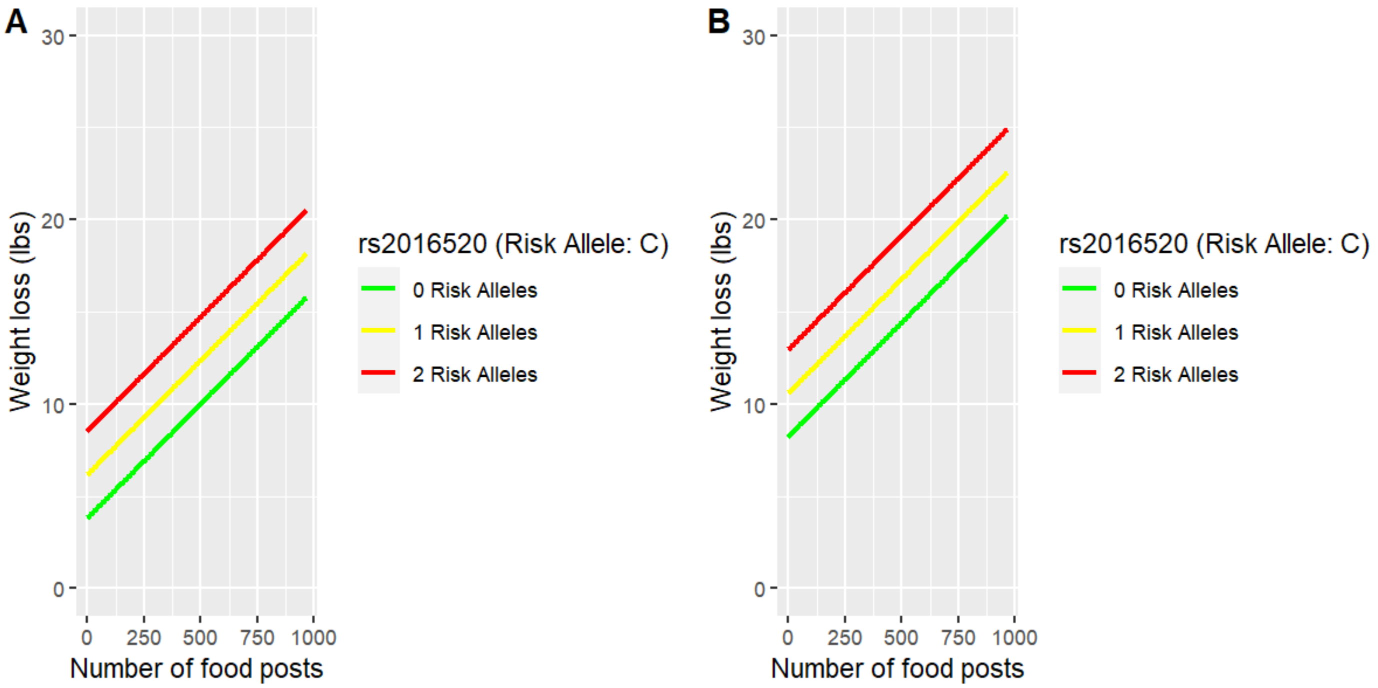

Figure S4: Weight loss (lb) vs number of food photos posted by rs2016520 (cholesterol SNP) in females (A) compared with males (B).

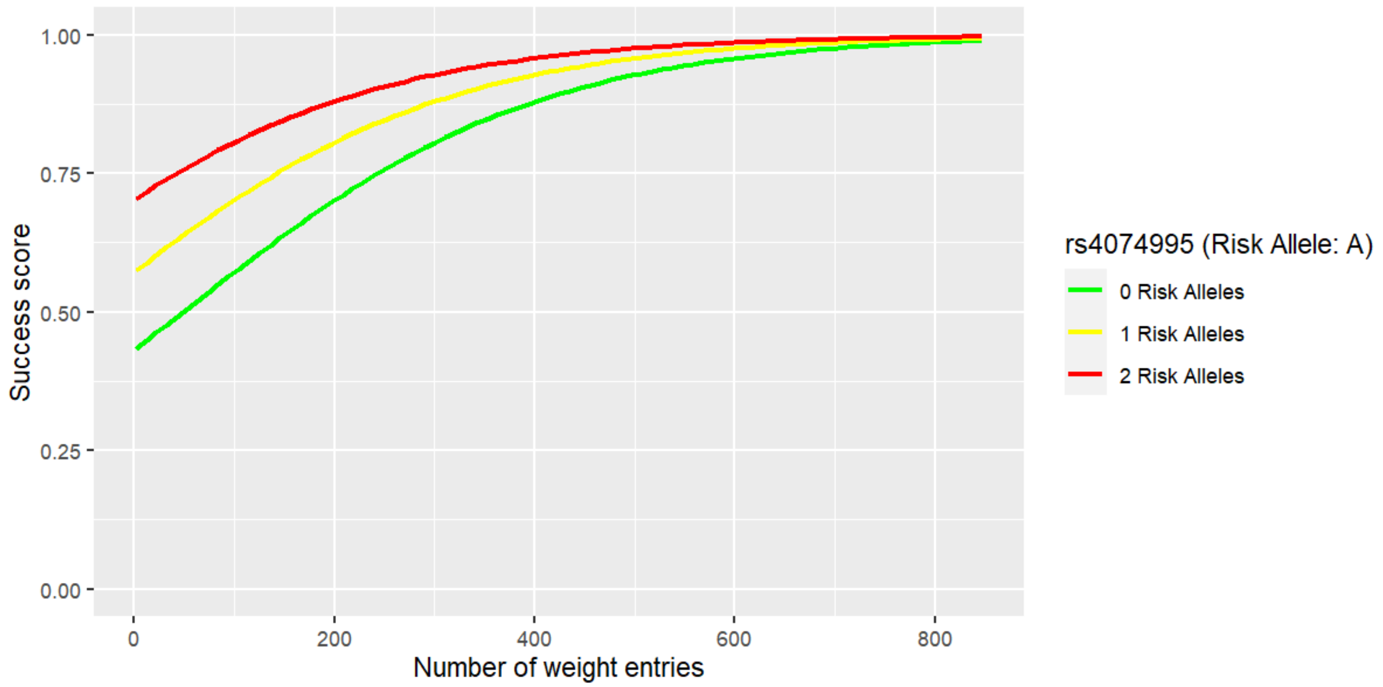

Figure S5: Weight loss success vs number of weight entries by rs4074995 (calcium-potassium SNP). The logistic regression success score is the likelihood of this model assigning the participant to the success class. Success score is depicted as a function of number of weight entries logged in the Digbi Health app (a behavioral engagement measure) by allele value of rs4074995. This SNP is varied over 0,1, and 2 risk alleles. All other SNPs in the model were held constant at their most frequent value.

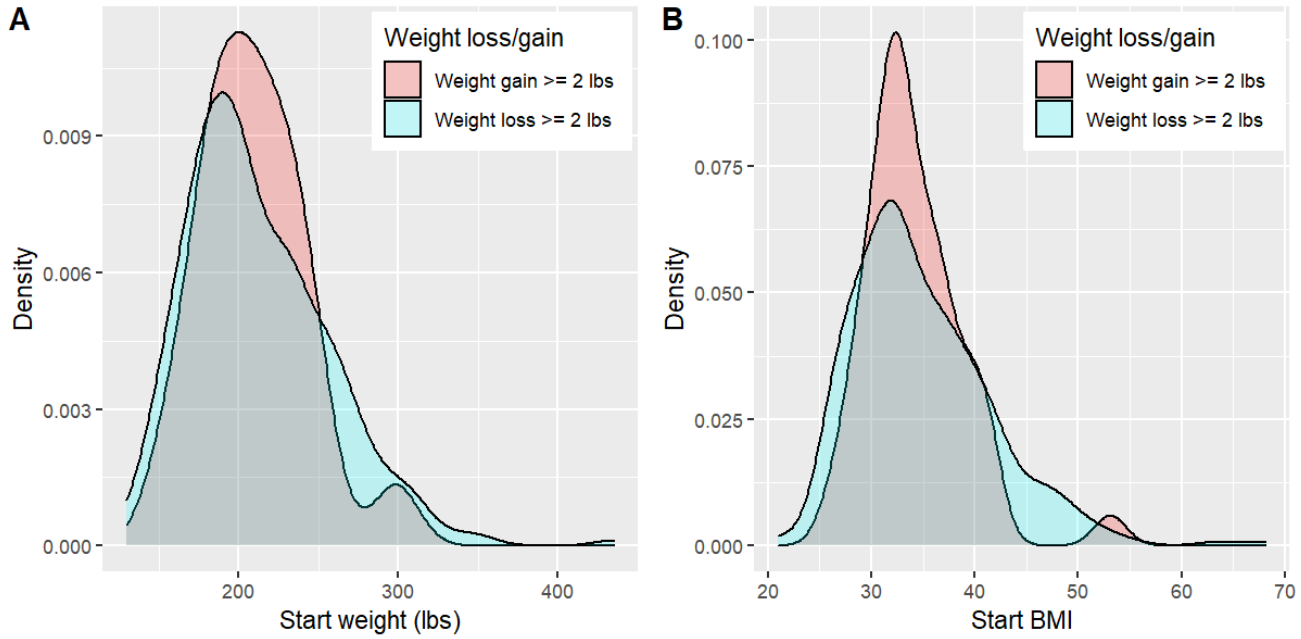

Figure S6: Baseline Weight Distributions are Similar for participants who gained versus lost weight. No statistical difference in means in A) start weight and B) start BMI, confirmed by Welch's two-sample t-test **(***P*=.64 and *P*=.42 respectively.) between subjects who lost (Blue) vs. gained (Red) weight. Less than 2 lbs gain or loss was considered negligible and excluded from this figure.

Table S6
Linear Model Weight Loss in Pounds WITHOUT Genomic Information

Variable	Estimate	*P*-value
Male Gender	-4.691200	*P*<.001
Starting BMI	0.175610	*P*=.006
Number of Food Photos Posted	0.013287	*P*<.001
Number of Coaching Sessions Completed	0.765527	*P*<.001

Table S7
Linear Model Weight Loss in Pounds WITH Genomic Information

Variable	Estimate	*P*-value
Number of coaching sessions	0.787908	*P*<.001
Male gender	-4.663601	*P*<.001
Number of food posts	0.012398	*P*<.001
rs2016520	2.362252	*P*<.001
Start BMI	0.199397	*P*=.001
rs10741657	-1.750832	*P*=.002
rs4074995	1.617627	*P*=.01
rs7138803	-1.682353	*P*=.008
rs9376026	1.715229	*P*=.002
rs236918	1.908988	*P*=.01
rs10246939	-1.118222	*P*=.047
rs1042713	1.437565	*P*=.02
rs2112347	1.310121	*P*=.02
rs2185570	1.859366	*P*=.04

Table S8
Linear Model Weight Loss % WITHOUT Genomic Information

Variable	Estimate	*P*-value
Male gender	-1.258215	*P*=.01
Number of food posts	0.006337	*P*<.001
Number of coaching sessions	0.371819	*P*<.001

Table S9
Linear Model Weight Loss % WITH Genomic Information

Variable	Estimate	*P*-value
Number of coaching sessions	0.366315	*P*<.001
Number of food posts	0.005971	*P*<.001
rs2016520	1.161539	*P*<.001
rs9376026	0.947039	*P*<.001
rs236918	1.232999	*P*<.001
rs10741657	-0.824197	*P*=.002
rs4074995	0.817030	*P*=.006
rs7138803	-0.824408	*P*=.006
rs1042713	0.834681	*P*=.004
Male gender	-1.182711	*P*=.01
rs2112347	0.767689	*P*=.005
rs7903146	0.593860	*P*=.04
rs17300539	1.088278	*P*=.04
rs1501299	-0.699880	*P*=.03

Table S10
Logistic Regression Model WITHOUT Genomic Information.

Variable	Odds Ratio	2.5%	97.5%
Number of weight entries logged	1.006	1.002	1.009
Number of coaching sessions completed	1.304	1.169	1.465

Table S11
Logistic Regression Model WITH Genomic Information.

Variable	Odds Ratio	2.5%	97.5%
rs2016520	1.985	1.134	3.595
rs7138803	0.534	0.318	0.872
rs10741657	0.617	0.400	0.937
rs4074995	1.765	1.106	2.878
rs9376026	1.804	1.167	2.85
rs1799931	0.293	0.097	0.822
Number of weight entries logged	1.006	1.002	1.009
Number of coaching sessions completed	1.356	1.200	1.546
